# Multivariate Analysis Coupled with M-SVM Classification for Lard Adulteration Detection in Meat Mixtures of Beef, Lamb, and Chicken Using FTIR Spectroscopy

**DOI:** 10.3390/foods10102405

**Published:** 2021-10-11

**Authors:** Muhammad Aadil Siddiqui, Mohd Haris Md Khir, Gunawan Witjaksono, Ali Shaan Manzoor Ghumman, Muhammad Junaid, Saeed Ahmed Magsi, Abdul Saboor

**Affiliations:** 1 Department of Electrical and Electronic Engineering, Universiti Teknologi Petronas, Seri Iskandar 32610, Malaysia; harisk@utp.edu.my (M.H.M.K.); saeed_19001716@utp.edu.my (S.A.M.); 2 BRI Research Institute, Jl. Harsono RM No. 2, Ragunan, Passsar Minggu, Jakarta 12550, Indonesia; gunawan.witjaksono@utp.edu.my; 3 Department of Chemical Engineering, Universiti Teknologi Petronas, Seri Iskandar 32610, Malaysia; ali_19001079@utp.edu.my; 4 Department of Electronics Engineering, FICT, BUITEMS, Quetta 87300, Pakistan; m.junaid@buitms.edu.pk; 5 High Performance Cloud Computing Centre (HPC), Universiti Teknologi Petronas, Seri Iskandar 32610, Malaysia; abdul_19001745@utp.edu.my

**Keywords:** food adulteration, halal authentication, Fourier transform infrared (FTIR) spectroscopy, principal component analysis (PCA), chemometric methods, multiclass support vector machine (M-SVM)

## Abstract

Adulteration of meat products is a delicate issue for people around the globe. The mixing of lard in meat causes a significant problem for end users who are sensitive to halal meat consumption. Due to the highly similar lipid profiles of meat species, the identification of adulteration becomes more difficult. Therefore, a comprehensive spectral detailing of meat species is required, which can boost the adulteration detection process. The experiment was conducted by distributing samples labeled as “Pure (80 samples)” and “Adulterated (90 samples)”. Lard was mixed with the ratio of 10–50% *v*/*v* with beef, lamb, and chicken samples to obtain adulterated samples. Functional groups were discovered for pure pork, and two regions of difference (RoD) at wavenumbers 1700–1800 cm^−1^ and 2800–3000 cm^−1^ were identified using absorbance values from the FTIR spectrum for all samples. The principal component analysis (PCA) described the studied adulteration using three principal components with an explained variance of 97.31%. The multiclass support vector machine (M-SVM) was trained to identify the sample class values as pure and adulterated clusters. The acquired overall classification accuracy for a cluster of pure samples was 81.25%, whereas when the adulteration ratio was above 10%, 71.21% overall accuracy was achieved for a group of adulterated samples. Beef and lamb samples for both adulterated and pure classes had the highest classification accuracy value of 85%, whereas chicken had the lowest value of 78% for each category. This paper introduces a comprehensive spectrum analysis for pure and adulterated samples of beef, chicken, lamb, and lard. Moreover, we present a rapid M-SVM model for an accurate classification of lard adulteration in different samples despite its low-level presence.

## 1. Introduction

The verification of authenticity and the detection of adulterants are critical aspects of food control, particularly in high-value items. As a measure of food quality and authenticity, laboratory data as well as chemical, physical, and visual pictures of foodstuffs are employed. The authenticity of the food is a major concern in the worldwide food industry; with the abundance of packaged food with a lengthy supply chain on the market, food authenticity is still an issue, as introduced by Spink and Mayor [1]. Nowadays, manual inspection, which is highly impacted by subjective variables, is nevertheless used frequently in quality evaluation. As a result, detecting pork in a variety of food items has become a major research topic in many countries, particularly in those where religious laws restrict the eating of pig products. Food adulterations may only financially impact a part of the population, but others may be more seriously affected [2,3,4] due to food poisoning, their religious views [5,6], etc. Some of the food tampering has been poisonous, for instance, such as the addition of sawdust to make white bread [7,8], the melamine adulteration of formula milk [7,9,10], the mixing of oil for engines with oil for human consumption in Spain [11]; some cases also involved the misrepresentation of food ingredients such as the UK horse meat issue in 2013 [12,13,14,15]. There are several ways of determining the provenance of animal species in meat products that are based on nucleic acid resources, commonly known as molecular techniques, which include DNA finger printing, PCR assays and PCR simple sequence repeat (PCR-SSR) [16,17], chromatographic techniques, isotopic techniques, vibrational and fluorescence spectroscopy, elemental techniques, nuclear magnetic resonance spectroscopy, sensory analysis, non-chromatographic mass spectrometry, immunological techniques, along with chemometrics and bioinformatics [18]. However, each methodology has its own set of drawbacks such as being costly, time-consuming, and inefficient, as well as requiring a wide range of equipment and making it difficult to understand the acquired data; moreover, most of these methods often require extensive sample preparation or are very susceptible to impurities. Unless all the protocols are strictly followed, they may lead to unpredictable outcomes. As a result, establishing a quick and reliable identification procedure to recognize meat species is critical. To address these restrictions, individuals have increasingly turned to spectroscopic methods in recent years. Fourier transform infrared spectroscopy (FTIR) has been widely used in the identification of agricultural commodities such as wine, olive oil, tea, and meat due to its quick and easy operation [19,20,21,22]. Research into food-authentication vibrational spectroscopy technologies today has been growing [22,23,24,25,26], partly because the sample preparation using the FTIR technique is relatively simple, results are relatively rapid, and this process is non-destructive in nature. The FTIR spectroscopic methods are thus fast becoming popular [27,28,29,30,31,32,33]. Some researchers have started to veer to Near Infrared (NIR) spectroscopy, mainly because its feasibility would open the possibilities of making the food authentication instrumentation set-up portable [28,29,30]. FTIR is quick and relatively inexpensive, with an easier sample preparation and a non-destructive process [18,19,24,34]. FTIR spectroscopy can distinguish meat and lard in meatball broth quickly and with high accuracy [19,21]; it has also been used with chocolate [24,34] and vegetable oils [22]. Table 1 presents the summary of methods and adulterants used in the literature, along with the multivariate techniques used for detecting the adulteration in different meat species. Therefore, the aim of this study was to utilize in-depth FTIR spectral analysis to improve the accuracy of lard adulteration detection by employing the classification of pure and adulterated samples combined with an M-SVM analysis for lard adulterated in mixtures of beef, lamb, and chicken. 

## 2. Materials and Methods

### 2.1. Meat Sample Collection

All meat samples were obtained from the local market at Seri Iskander in Malaysia. After that, the meat was washed with purified water and cut into small parts (1 cm × 1 cm) and held at −10 °C. Total samples were then divided into two different classes, as pure and adulterated. There were 80 pure and 90 adulterated samples produced for the spectral analysis. The sample preparation was designed to be straightforward, with no extra chemical substances used. Beef, lamb, and chicken loin cuts were used, and all pork was lean meat taken from chops.

### 2.2. Extraction Procedure and Sample Distribution

Lard and other animal body fats from meat such as chicken fat, beef fat, and mutton fat were extracted according to the method stated by [34], with little variation. All samples were gradually heated from 50 °C to 150 °C for 45 min until the fat was extracted from all the samples on the petri dish. The discharged fat was then filtered as the concentration contained solid minute particles. Moreover, samples were centrifuged at 3000 rpm for 20 min and filtered through Whatman filter paper. Pure fats produced by the extraction process were then used to make adulterated samples. All the chemicals used in this experiment were of analytical consistency. Pure and adulterated fats were then analyzed using FTIR spectroscopy. The instrument used was Frontier FT-IR by PerkinElmer. The optical system with KBr beam splitter was used to enable quality data collection over a range of 8300–350 cm^−1^ at a best resolution of 0.4 cm. The resulting spectrum contained 2500 continuous values for one sample, with intervals of 0.8 cm^−1^. To guarantee that there was no major fluctuation between each spectra scanned, each spectrum was recorded at the same temperature. This procedure was required to remove any uncontrolled ambient influences on the instrument and the sample.

### 2.3. Spectral Data Pre-Processing

Smoothing and normal variate transformation (SNV) were used as spectrum pre-processing approaches in this investigation. The reflectance spectra were smoothed by Savitzky-Golay smoothing using a second-order polynomial and a 5-point window to eliminate the random disturbances caused by the system’s internal components. SNV was used to adjust for scatter effects and reduce slope variation. The Savitzky-Golay smoothing filter was used to increase the precision of the data without distorting the signal tendency.

### 2.4. Preparing Mixture Samples

Lard was mixed with body fats of lamb, beef, and chicken to obtain a series of standard or trained sets of 80 pure and 90 adulterated samples containing 10–50% *v*/*v* of lard in lamb, beef, and chicken samples, as shown in Table 2. The following method is according to Rohman et al. [23]. We prepared six pieces for each combination of lard mixed with a defined percentage of lamb, chicken, and beef, with pork in the proportion of 10, 20, 30, 40, and 50%, whereas B-50%, L-50%, and C-50% represent a 50-50 ratio of pork with beef, lamb, and chicken, respectively; meanwhile, B-90%, L-90%, and C-90% indicate 10% lard with 90% of the respective species. The detailed distribution of samples is presented in Table 3.

## 3. Results and Discussion

After a careful process of sample-making and data pre-processing, the obtained spectrum for both pure and adulterated samples was analyzed separately. The developed workflow for further investigating the lard adulteration was carried out using a three-stage process. In the first stage, identification of functional groups in lard samples without any contamination was made. Secondly, pure spectral samples of beef, lamb, chicken, and lard were analyzed by overlapping the spectrums and identifying the region of difference (RoD) for highly significant regions. Moreover, the profiling of adulterated samples with the percentage difference for beef, lamb, and chicken was also carried out. After spectral analysis, the third and final stage combined the multivariate analysis with M-SVM classification for both pure and adulterated samples separately. Samples were divided into two classes, ‘Haram (lard)’ and ‘Halal (chicken, lamb, and beef)’, for M-SVM classification.

### 3.1. FTIR Spectra Analysis of Pure Samples

**Figure 1:**
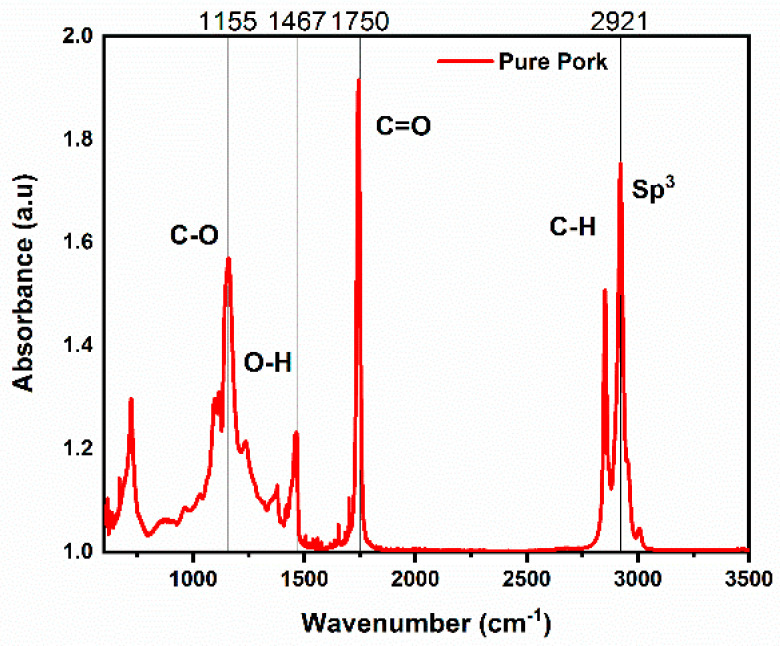
Spectrum analysis of pure pork identifying the frequencies for functional group vibrations.

Amid the four different meat fats, the pure lard used in this study was evaluated and analyzed separately using FTIR spectroscopy. The peak is shown in Figure 1 approximately at wavenumber 2921 cm^−1^, which was due to the tensile vibration of C-H (Sp^3^) in = C-H cis. The functional group-CH_2_ provided peaks at wavenumber 2853 cm^−1^ consecutively as result of asymmetrical and symmetrical vibration. The peak showed the triglyceride ester carbonyl (C=O) group at wavenumber 1750 cm^−1^.

In the fingerprint region, vibrations of the stretching mode from the C-O group in esters were detected at wavenumber 1155 cm^−1^, while at wavenumber 1467 cm^−1^ the bending vibrations of the CH_2_ and CH_3_ aliphatic groups were detected, as shown in Figure 1. Table 4 shows the details of wavenumber and the associated vibration of functional groups for the pure lard sample.

**Figure 2:**
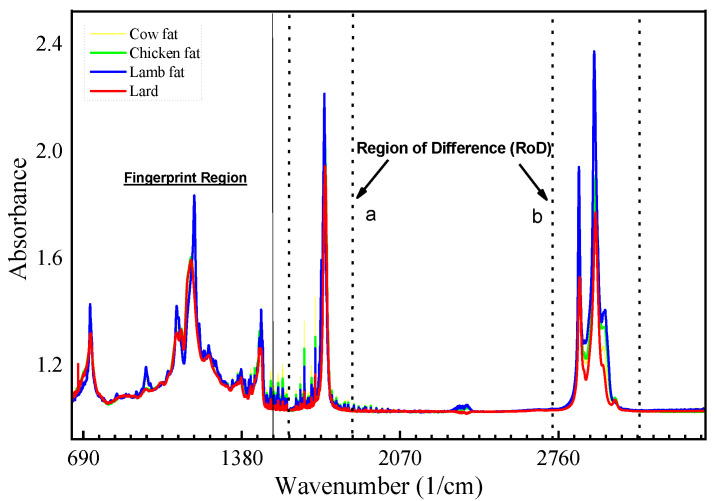
Overlapped spectrum from FTIR covering 3500–650 cm^−1^, representing the fingerprint and functional group regions for pure samples of beef, lamb, lard, and chicken, with identification of potential regions of difference (RoD).

**Figure 3:**
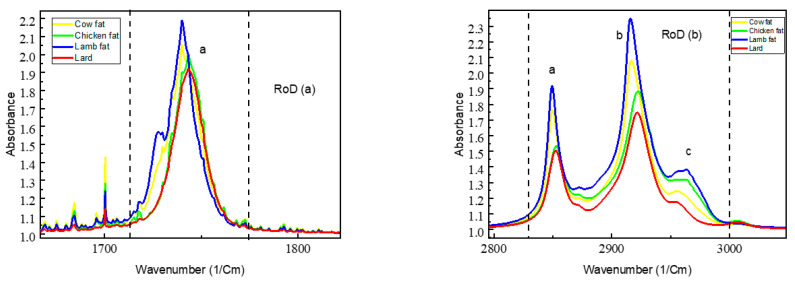
RoD(a) Region of difference peaks zoomed in at wavenumber 1700–1800 cm^−1^ showing the absorbance value for pure samples denoted by a; lard has the lowest value among all samples. RoD(b) Zoomed-in peaks at wavenumber 2800–3000 cm^−1^ where peaks denoted as a, b, and c represent potential regions with difference in absorbance values for all samples.

Figure 2 below shows the FTIR spectra of pure samples overlapped for the identification of wavenumbers, with associated peaks identified as the region of difference (RoD) along with the fingerprint region. This spectrum can be divided into three regions to make the analysis convenient: the first region range is at wavenumber 3000–2500 cm^−1^, the second region range is 2000–2500 cm^−1^, the third region range is 1500–2000 cm^−1^, and to conclude, the fingerprint region range is at wavenumber 1500–500 cm^−1^. Two separate regions are highlighted with dotted lines (a and b), with the overlapping of pure samples for all species, as indicated in Figure 2, where the change in absorbance values is highly prominent; wavenumbers associated with these two regions are in the spectrum ranges of 1700–1800 cm^−1^ for RoD(a) and 2800–3000 cm^−1^ for RoD(b) respectively as shown in Figure 3. The FTIR spectra of all the lipids obtained from different species were combined and overlapped.

**Figure 4:**
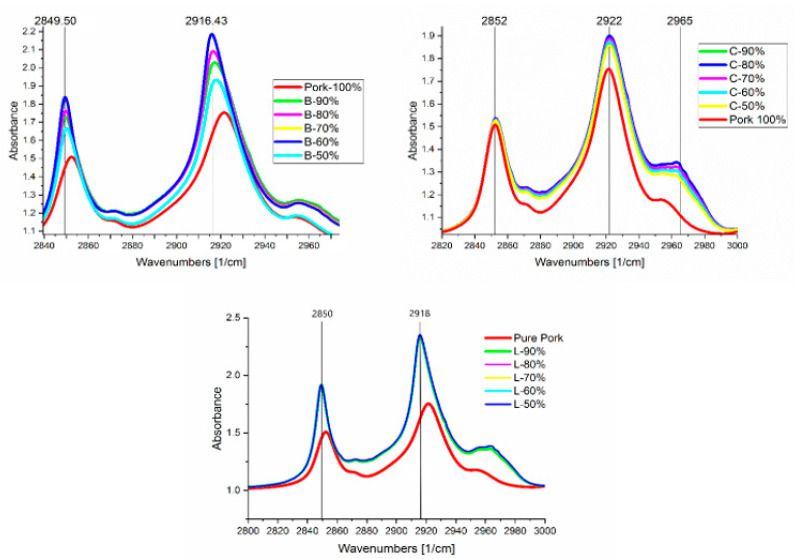
Peaks zoomed-in for adulterated samples overlapped with pure pork, for beef (B-50% to B-90%), lamb (L-50% to L-90%), and chicken (C-50% to C-90%), with associated adulteration ratio and associated peaks for RoD(a) and RoD(b).

As the value for the adulteration of lard increases for both beef and chicken, the absorbance values merge with the lard, showing high contrast compared to lamb samples, which indicates negligible change when lard is mixed. This is clearly visible in the spectral analysis shown in Figure 4 for all the adulterated samples. The absorbance values in the region of RoD(b) are carefully analyzed, where the adulteration of lard can potentially be detected. This is shown in Table 5. On the other hand, beef samples are highly prone, and lard is detectable because of the significant change in absorbance value at the region of 2800–3000 cm^−1^ in the spectrum, specifically at RoD(b) a and b, which represent regions at 2840–2860 and 2900–2940 cm^−1^, respectively. Table 5 lists all the absorbance values at the peaks of RoD(b) in Figure 2; the percentage difference is calculated with respect to lard for peak absorbance in regions with high significance.

The highest proximity of absorbance values to pure lard can be seen in the samples of B-50%, C-90%, C-80%, and C-50%, for both regions RoD(b)-a and RoD(b)-b. At the same time, adulterated beef shows a pattern of variation according to the adulteration percentage of lard. Beef samples with 10% adulteration (B-90%) have an approximate percentage difference of 7–14%, while beef with 50% adulteration (B-50%) shows approximately 3–8% change for both regions. All samples containing adulterated chicken from C-50% to C-90% show the lowest percentage difference as compared to lamb and beef. This reveals the highest similarity to be between chicken and lard, which could present some difficulty in detecting the adulteration of lard in chicken irrespective of the percentage mixing. Moreover, adulterated lamb samples depict minor variation in absorbance values throughout the mixing samples (L-50% to L-90%) and have the highest percentage difference as compared to pure lard.

### 3.2. Results of Principal Component Analysis

Pure lard, along with other samples of beef, chicken, and lamb, was classified using the chemometric of PCA. PCA is used to reduce the dimension of the spectral signal. The wavenumber regions for PCA were also optimized. To confirm the separation based on adulterant type, the raw data (eigenvectors of the covariance matrix) was subjected to principal component analysis (PCA). Further explanation on PCA is at Appendix A.1

**Figure 5:**
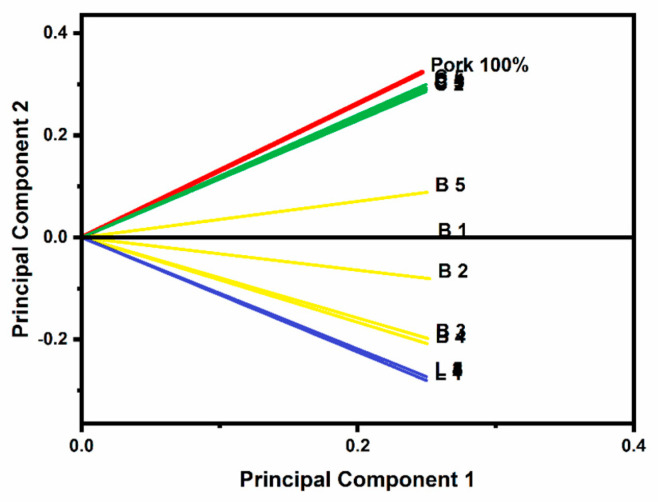
Principal component analysis plot showing the similarity between pork, chicken, lamb, and beef samples with adulterated mixtures. C1–C5 (10–50% Pork), B1–B5 (10–50% Pork), L1–L5 (10–50% Pork).

It is possible to observe a distinct split depending on the level of adulteration by showing the scores of the first two main components (Figure 5), which represent 99.36 percent of data variance. Only a little amount of overlap exists between the chicken samples that have been tainted with pork. The selection of wavenumbers was based on their ability to provide a useful classification between samples, as seen in Figure 5. The PCA plot showed clusters of samples based on their similarity with the first main component (PC1) and the second main component (PC2), which provided a good separation between the lamb, beef, and pork groups but was unable to separate pork and chicken. The percentage (%) variability of PC1 and PC2 was 97.31% and 2.05%, respectively. PC1 comprised the most variation of the data, as shown in Table 6.

**Figure 6:**
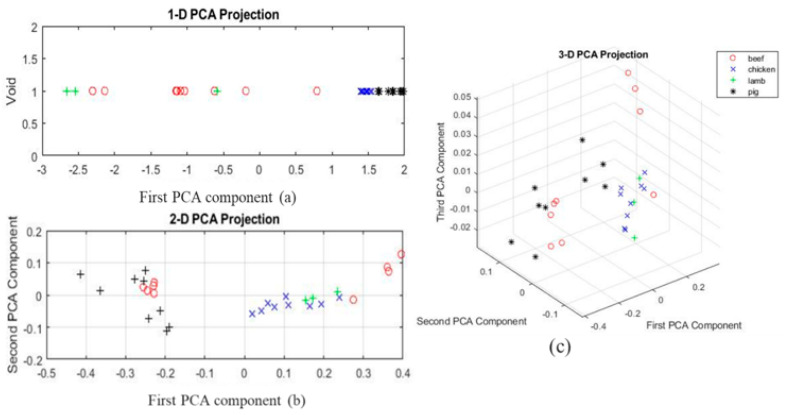
(**a**) Pure samples of all the species using a one-dimensional projection for first principal component. (**b**,**c**) Principal component analysis of the two and three-dimensional projections of adulterated samples of pork (*), chicken (×), lamb (+), and beef (O), showing the clustering.

The FTIR spectra of the pure pork sample were compared with those of adulterated beef, chicken, and lamb. Three dimensional plots are shown in Figure 6. The PCA analysis shows the PCA projection divided into three dimensions for better analysis.

Figure 6a shows the distribution of samples across the first principal component using 1D spectra of the pure samples for beef, lamb, chicken, and pork, where chicken and pork samples overlap and correlate highly coupled values of absorbance with similar wavenumbers. At the same time, Figure 6b depicts the samples at PC1 and PC2 using 2D representation for all the adulterated species. Figure 6c combines all the three principal components using 3D for all the adulterated samples. The regions in these figures are separated based on the adulteration quantity, starting with slightly mixed, i.e., 10%, to highly adulterated, i.e., 50%. In the first projection, the plotted points representing the samples of chicken, beef, and lamb are scattered, and they are far from the pork group. The closer the dots of chicken, beef, and lamb are to the pork samples, the more significant the quantity of lard is in pure samples.

### 3.3. Multiclass Support Vector Machine Classification

**Figure 7:**
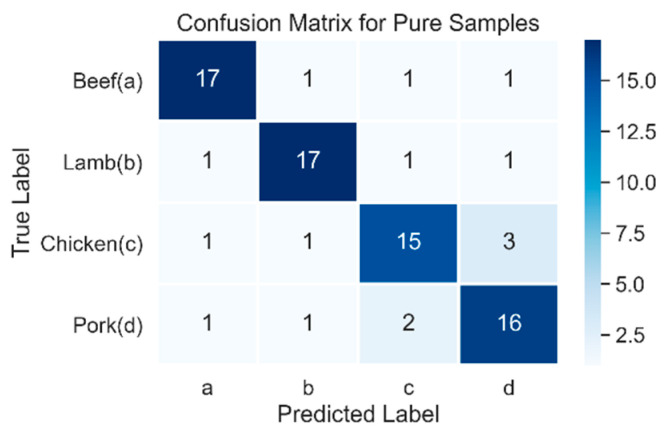
Heatmap confusion matrix of multiclass classification for pure samples of beef, chicken, lamb, and pork showing the predicted and true labels.

The data obtained from the previous processes were divided into testing data (30%) and training data (70%), and subsequently evaluated with the classification model. The data acquired from the FTIR spectroscope was analyzed using the scikit-learn machine learning library in Python. The radial basis function (RBF) was used as the kernel function of SVM using the grid search method. To add an extra validation step to our model, we used the confusion matrix for both multiclass datasets, as shown in Table 7 and Table 8. The confusion matrix projects the true data against predicted data. In our study, we divided the problem into two different sections: one identified pure samples correctly, and the other predicted the adulterated samples. The learning rate was 0.0001, and the regularization parameter λ was set to 1/epochs. Table 7 illustrates the user, producer, and overall accuracy of the pure samples data set. Details of the SVM is explained at Appendix A.2. Pure samples of beef and lamb using optimal parameters produced the highest accuracy (85%) among all the samples. Furthermore, pure samples of chicken had the lowest accuracy of 75%, whereas pure pork was significantly better than chicken, with 80% accuracy. Moreover, Figure 7 shows a confusion matrix using a 10-fold cross-validation for the pure samples where the a, b, and c rows represent the true label; meanwhile, according to the model prediction, the a, b, and c columns represent the number of predicted sets for each respective class.

The predicted labels for pure samples shown in Figure 7 misclassified three samples of pure chicken as pure pork, while two samples of pure pork were falsely labeled as chicken. Moreover, beef and lamb both had three label misclassifications, one for each species of meat.

**Figure 8:**
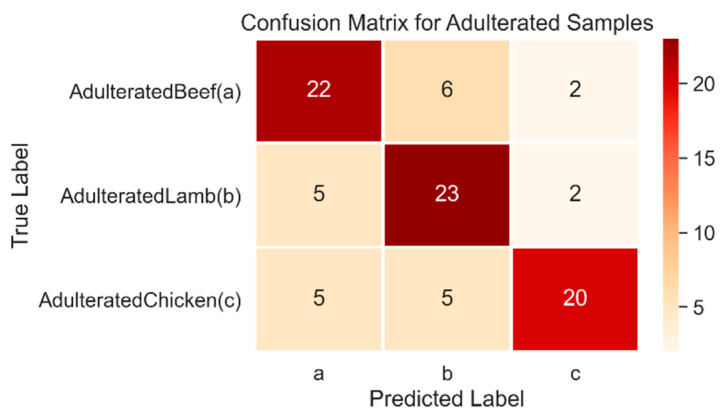
Heatmap confusion matrix of the multiclass SVM classifier for adulterated samples of beef, chicken, and lamb.

Table 8 shows the confusion matrix for the multiclass SVM of adulterated data samples. The adulterated data set contained all the samples that were adulterated with different proportions of lard. The AdulteratedBeef sample included samples with a *v*/*v* ratio from B-50% to B-90%. The producer accuracy was highest for AdulteratedLamb at 76.6%, whereas AdulteratedBeef had the second-highest value of 73.3%. The spectrum of lamb had no change in absorbance value when it was adulterated, irrespective of the adulteration ratio, which was also validated by the SVM classifier by getting the maximum number of correctly classified labels, as shown in Figure 8.

AdulteratedChicken samples, with 20 correctly classified samples, produced the lowest precision accuracy of 66% due to its high variation in absorbance values, as shown in Figure 8.

## 4. Conclusions

FTIR spectroscopy, coupled with the multivariate and M-SVM methods, seems to be an efficient and rapid technique for the discrimination of lard from other meat samples. In this paper, we demonstrated the identification and discrimination of lard from beef, chicken, and lamb fats in meat mixtures. FTIR spectral analysis in combination with Principal Component Analysis (PCA) and M-SVM have shown that pure lard fat has unique peaks that can distinguish the pork from beef, chicken, and lamb meat at wavenumbers 1155 cm^−1^, 1467 cm^−1^, 1750 cm^−1^, and 2921 cm^−1^. The absorbance values indicate a direct correlation between lard and other species. The PCA results show that adulteration in chicken meat is positively correlated with pork meat, while lamb is negatively correlated with respect to lard. The SVM model produced an overall prediction accuracy of 81.25% for pure samples, and for adulterated samples, it showed a 72.2% prediction accuracy. The overall accuracy was computed using the sensitivity and precision values. The model accurately classified the pure samples better than the adulterated samples due to a smaller number of samples and the minimalistic difference in absorbance values of the spectrum. Thus, this study has the potential to establish as a rapid method for halal authentication and could revolutionize the in-line quality control in the meat industry. For future work, the FTIR profiles for pure and adulterated samples can be increased, and deep learning may be applied for detecting an adulteration quantity of less than 10%.

## Figures and Tables

**Table 1:** Summary of food analyses using multivariate techniques with infrared spectroscopy for the detection of meat species adulteration [35,36,37,38,39,40].

Method	Meat Adulterant	Analysis Technique
Fourier Transform Infrared Spectroscopy	Palm Oil with Chicken Fat	Linear Discriminant Analysis
E-Nose	Lard, Chicken, and Beef	K-Nearest Neighbors algorithm (KNN), Support Vector Machine (SVM)
Fourier Transform Infrared Spectroscopy	Beef Jerky with pork	LDA, SIMCA, and SVM
Fourier Transform Infrared Spectroscopy	Lard, Mutton, and Cow	PLS Regression
Raman Spectroscopy	Beef and Horsemeat	PCA
Fourier Transform Infrared Spectroscopy	Lard and Palm Oil	PLS
Fourier Transform Infrared Spectroscopy	Lard, Beef Meatballs	PCA and PLS
Fourier Transform Infrared Spectroscopy	Lard in Palm Oil	PCA and PLS

**Table 2:** Distribution of adulterated and pure samples along with the number of pieces produced and spectra obtained for individual species.

Meat Specie	Number of Pieces	Number of Samples Obtained		Number of Spectra Obtained
		Pure Samples	Adulterated Samples (*v*/*v*)	
Beef	20	10 × 2 = 20	15 × 2 = 30	50
Lamb	20	10 × 2 = 20	15 × 2 = 30	50
Pork Chicken	20 20	10 × 2 = 20 10 × 2 = 20	- 15 × 2 = 30	20 50
Total	80	80	90	170

**Table 3:** Composition of adulterated samples with the ratio of lard mixed with samples of beef, lamb, and chicken, represented by their initials (Lamb: L-90% to L50%, Beef: B-90% to B-50%, Chicken: C-90% to C-50%).

Mixture Samples Label	Pork (*v*/*v*)	Lamb (*v*/*v*)	Beef (*v*/*v*)	Chicken (*v*/*v*)	Number of Samples
L-90%	10%	90%	-	-	6
L-80%	20%	80%	-	-	6
L-70%	30%	70%	-	-	6
L-60%	40%	60%	-	-	6
L-50%	50%	50%	-	-	6
B-90%	10%	-	90%	-	6
B-80%	20%	-	80%	-	6
B-70%	30%	-	70%	-	6
B-60%	40%	-	60%	-	6
B-50%	50%	-	50%	-	6
C-90%	10%	-	-	90%	6
C-80%	20%	-	-	80%	6
C-70%	30%	-	-	70%	6
C-60%	40%	-	-	60%	6
C-50%	50%	-	-	50%	6
Total Mixture Samples					90

**Table 4:** Functional group and associated mode of vibration for pure lard.

Frequency (cm^−1^)	Functional Group Vibration
1155	Vibrations of stretching mode from the C-O group in esters
1467	Bending vibrations of the CH_2_ and CH_3_ aliphatic groups
1750	Carbonyl (C=O) functional group of the ester linkage of triacylglycerol
2921	Asymmetrical or symmetrical stretching methylene (-CH_2_) band vibration

**Table 5:** Absorbance values and percentage difference with respect to lard for adulterated samples of beef, lamb, and chicken in the region of RoD(b) at the highly significant region of 2800–3000 cm^−1^.

Species Type	Sample	Absorbance Value at RoD(b)-a	Absorbance Value at RoD(b)-b	Percentage Difference w.r.t Pork	
**Pure Lard**	Pork-100%	1.5963	1.75306	RoD(b)-a	RoD(b)-b
**Adulterated Beef**	B-50%	1.6580	1.9154	3.79%	8.85%
	B-60%	1.8357	2.1793	13.95%	21.67%
	B-70%	1.8310	2.1784	13.69%	21.63%
	B-80%	1.7611	2.0906	9.81%	17.56%
	B-90%	1.7262	2.0227	7.81%	14.28%
**Adulterated Chicken**	C-50%	1.5256	1.8577	4.52%	5.79%
	C-60%	1.5289	1.8737	4.31%	6.65%
	C-70%	1.5312	1.8868	4.16%	7.34%
		1.5358	1.8995	3.86%	8.01%
	C-90%	1.5358	1.8995	3.86%	8.01%
**Adulterated Lamb**	L-50%	1.8739	2.2576	15.99%	25.15%
	L-60%	1.8739	2.2576	15.99%	25.15%
	L-70%	1.8739	2.2576	15.99%	25.15%
	L-80%	1.8739	2.2576	15.99%	25.15%
	L-90%	1.8710	2.2396	15.84%	24.37%

**Table 6:** Percentage of variance for each PCA component contributing to the variation of the classification.

Principal Component	Variance Contribution
PC1	97.31%
PC2	2.05%
PC3	0.64%

**Table 7:** Sensitivity, precision, and classification accuracy for pure samples of beef, lamb, chicken, and pork.

Classified as	User Accuracy (Sensitivity)	Producer Accuracy (Precision)	Overall Accuracy
Beef	85%	85.00%	81.25%
Lamb	85%	85.00%	
Chicken	78%	75.00%	
Pork	76%	80.00%	

**Table 8:** Sensitivity, precision, and classification accuracy for adulterated samples of beef, chicken, and lamb.

Classified as	User Accuracy (Sensitivity)	Producer Accuracy (Precision)	Overall Accuracy
a = AdulteratedBeef	68.86%	73.33%	72.2%
b = AdulteratedLamb	67.19%	76.66%	
c = AdulteratedChicken	83.20%	66.00%	

## Data Availability

Data presented in this study is available at request from the corresponding author.

## Appendix A

### Appendix A.1. Principal Component Analysis

Principal Component Analysis (PCA) is a statistical technique that is particularly useful in reducing observations that have many dimensions. This technique consists of transforming dimensions of a dataset into a new but smaller set of uncorrelated dimensions called principal components (PCs). An array of ($q_{ij}$) values can be normalized using the equation below:

(A1) $$X_{ij}=q_{ij}-{\overset{‐}{q}}_{j}$$

The data given to us is the array element data corresponding to the variable $X_{ij}$, and the mean value of the variable ${\overset{‐}{q}}_{j}$. Then, using the new dataset array, a correlation matrix is constructed so that information about how the variables in the dataset are correlated can be obtained. To create our new correlation matrix *X* with the new correlation coefficients $X_{ij}$, the following formula is used:

(A2) $$R=X^{T}•X$$

Only the principal components that explain the greatest amount of data in the original are determined using the equation below:

(A3) S=V•Q

where *S* is the matrix data, known as Score; *V* is the eigenvectors; and *Q* is the original data array. The matrix *S* (Score) will now represent the data in a way that each column represents the projection of the initial data *Q*.

### Appendix A.2. Support Vector Machine Classification

Most machine learning techniques have been created and statistically verified for linearly separable data. For the reduction of dimensionality, linear classifiers such as Support Vector Machines (SVMs) or the (conventional) Principal Component Analysis (PCA) are common examples. However, to efficiently accomplish tasks involving pattern analysis and discovery, most real-world data require non-linear approaches. By incorporating the kernel trick, the SVM approach has improved over time. To detect a pattern in non-linear separable data, the kernel method effectively translates the input data to higher dimensions. When the training data has many variables in comparison to the number of observations, SVMs are an excellent classification approach. In SVM, every sample x that consists of n variables is treated as an n-dimensional vector. Prediction performance can be assessed using the following three indicators: sensitivity (User Accuracy), precision (Producer Accuracy), and overall accuracy. Precision is the proportion of appropriately positive labels produced by our software to all positive labels produced. The ratio of the exactly positive labels identified by our algorithm to all positive labels is referred to as sensitivity. Accuracy is the proportion of correctly categorized topics to the total number of issues. Equations (A4)–(A6) present the formula for Precision, Accuracy, and Sensitivity.

(A4) $$Sensitivity=\;\frac{True\;Positive\;}{Predicted\;Results}$$

(A5) $$Precision=\;\;\frac{True\;Positive}{Actual\;Results}$$

(A6) $$Overall\;Accuracy=\;\frac{True\;Positive+True\;Negative}{Total}$$
